# Choroidal origin of endogenous *Candida* endophthalmitis

**DOI:** 10.1186/s12886-020-01540-8

**Published:** 2020-07-13

**Authors:** Mark P. Breazzano

**Affiliations:** 1 Edward S. Harkness Eye Institute, Department of Ophthalmology, Columbia University Irving Medical Center, New York-Presbyterian Hospital, 635 W 165th St, New York, NY 10032 USA; 2 Department of Ophthalmology, New York University School of Medicine, New York University Langone Health, New York, NY USA; 3 Manhattan Eye, Ear, and Throat Hospital, Lenox Hill Hospital, Northwell Health, New York, NY USA; 4 Wilmer Eye Institute, Johns Hopkins University School of Medicine, Johns Hopkins Hospital, Baltimore, MD USA

**Keywords:** Endogenous *Candida* endophthalmitis, Spectral-domain optical coherence tomography, Artifact, Choroid

## Abstract

Endogenous *Candida* endophthalmitis (ECE) has been established with microscopic histopathology, both by autopsy and experimentation, to primarily originate from and involve the choroid. Zhuang et al. examined a series of patients with ECE using spectral-domain optical coherence tomography (SD-OCT) imaging and present a new classification scheme. The authors conclude the majority of lesions are primarily retinal in location without report of choroidal involvement. This discrepancy may be explained by posterior shadowing artifact and lack of discernment from associated retinal findings like infarction. These considerations are necessary in reviewing SD-OCT, characterizing ECE, and proposing new classification systems.

## Main text

I studied the paper with interest by Zhuang et al. who examine a large series of endogenous *Candida* endophthalmitis (ECE) using impressive spectral-domain optical coherence tomography (SD-OCT) imaging [1]. They classify lesions based on observed location, and conclude that they are primarily retinal in the majority, 12/17 (70%). However, these authors have not sufficiently demonstrated retinal origin for a disease process that has been histologically established to primarily begin in the choroid [2–5]. Though a powerful tool, SD-OCT has limitations [6–8] which can mislead the examiner as illustrated in this study [1] and another before it [9], with misinterpretation of results. Understanding the pathogenesis of ECE as a primarily chorioretinal process, rather than retinal, is critical given important ramifications with clinical course, prognosis, and treatment [2, 10, 11].

Microscopic histopathology from autopsy has repeatedly shown that typical ECE foci localize to the inner choroid, which extend through Bruch’s membrane, the retina, and eventually the vitreous [2–4]. Although occasional isolated retinal lesions can be identified (approximately 14%) [4, 8], it remains unclear if these are always seeds from the retinal vasculature. Experimentally infected rabbits have also confirmed the primarily inner choroidal localization of *Candida* lesions [5]. These findings are consistent with experience from numerous metastatic processes for both infection and malignancy; while some can arise from retinal circulation, most have predilection for seeding through choroidal circulation given its inherently greater blood flow [12].

**Fig. 1:**
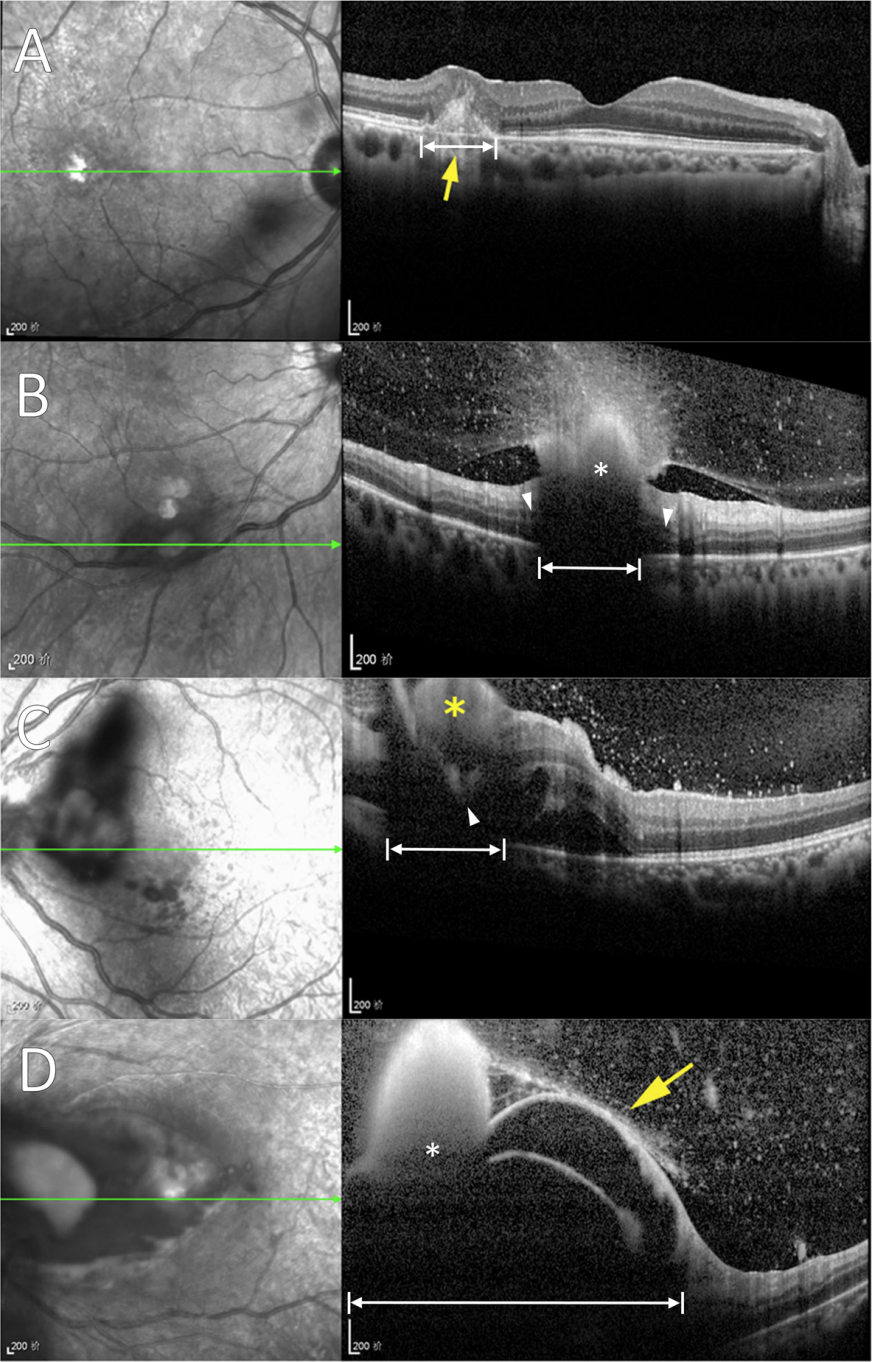
Imaging of endogenous Candida endophthalmitis (ECE) demonstrated by Zhuang et al. [1], now with labeling overlay (white markings). These examples of ECE (classified as “retinal” lesion types 1–4, respectively) demonstrate primary choroidal involvement (**a**), and shadowing that cannot establish a retinal origin or exclude choroidal origin (**b**-**d**). **a** Disrupted architecture of inner choroid (double arrow) extending into the overlying retinal pigment epithelium and neurosensory retina is consistent with a primarily choroidal infiltrative and inflammatory process. **b** Posterior shadowing (double arrow) is found approximate in size to the overlying, anterior aspect of a large hyperreflective structure (*). Lesion origin cannot be determined because the view of the choroid and outer retina is overshadowed (double arrows), but cystic changes are apparent in outer retina adjacent to the edges of shadowing (arrowheads) which suggest a possibly deep lesion origin. **c** Posterior extent of hyperreflectivity is seen extending at least into deep retina (arrowhead), surrounded by neurosensory retinal detachment and extensive shadowing that completely obscures underlying choroid (double arrows). **d** Large hyperreflective mass (*) with florid inner retinoschisis and secondary shadowing (double arrows) blocks choroidal visualization

The lack of findings in the choroid is important to recognize in this study by Zhuang et al. [1]. The discrepancy here of SD-OCT interpretation with prior histopathology can be attributed to oversight of a crucial finding. Posterior shadowing is a well-established artifact for many OCT modalities [6–8], and accounts for the signal void posterior to the reflective, anterior surfaces of the ECE lesions (Fig. 1). Without adequate visualization of the choroid from this artefactual shadowing, the subsequent classification of lesions based solely on retinal location dismisses this established, primary anatomical origin for these ECE lesions. Although the authors do mention their “type 1” retina lesion is similar to previously described chorioretinal lesions and refer to it as “subretinal” [1], this proposed classification elicits confusion as it implies the choroid is secondary or possibly uninvolved in ECE pathophysiology.

This distinction is critical for understanding the origin of intraocular involvement of *Candida* bloodstream infections and has ramifications for management. Even when whitish abnormalities confined to the retina are identified clinically, it can be challenging to differentiate them from other similar findings like infarction with OCT [8]. This limitation explains overestimation of 6/15 (40%) true *Candida* lesions arising from the retina in a recent SD-OCT study [9]. The number 12/17 (70%) here appears further inflated [1], probably because of additional overestimation from shadowing artifact.

Zhuang et al. imply that invasive intervention (intravitreal antifungal injection and/or vitrectomy) is necessary for all ECE, however, have not provided sufficient evidence [1]. Previous studies have demonstrated efficacy of systemic antifungal therapy alone (ideally with exchange of indwelling catheters) in many cases of ECE [2, 10]. As ECE primarily emanates from the choroid (outside of the blood-outer retinal barrier), relatively mild lesions with minimal vitreous haze (Fig. 1) are known to respond with this more conservative management without the risk of invasive intervention [2, 10]. Surgical risk may be further compounded in this patient population, as multiple pre-existing comorbidities are often present [10].

In conclusion, this study provides spectacular SD-OCT imaging examples of ECE, but caution should be advised with over-interpretation of the results through its proposed classification system and treatment strategy. New imaging results, then, should be presented in the context of established, pre-existing knowledge: the primarily choroidal origin of ECE, and the efficacy of treating ECE (the underlying *Candida* bloodstream infection) in certain cases that may not necessarily require invasive approaches.

## Data Availability

Not applicable.

## Abbreviations

ECE: Endogenous *Candida* endophthalmitis
SD-OCT: Spectral-domain optical coherence tomography
